# Nanoparticles of Antroquinonol-Rich Extract from Solid-State-Cultured *Antrodia cinnamomea* Improve Reproductive Function in Diabetic Male Rats

**DOI:** 10.2147/IJN.S252885

**Published:** 2020-06-15

**Authors:** Zwe-Ling Kong, Jia-Ling He, Sabri Sudirman, Mao-Tien Kuo, Song Miao, Ke-Liang B Chang, David Tsou

**Affiliations:** 1 Department of Food Science, National Taiwan Ocean University, Keelung City, Taiwan; 2 Fisheries Product Technology, Faculty of Agriculture, Universitas Sriwijaya, Palembang, Ogan Ilir Regency, Indonesia; 3 Lantyng Biotech, Co. Ltd., Taipei City, Taiwan; 4 Teagasc Food Research Center, Moorepark, Co. Cork, Ireland

**Keywords:** *Antrodia cinnamomea*, antroquinonol, diabetes, nanoparticles, male reproduction

## Abstract

**Purpose:**

To characterize the nanoparticle of antroquinonol from *A. cinnamomea* and its ameliorative effects on the reproductive dysfunction in the diabetic male rat.

**Material and Methods:**

The chitosan-silicate nanoparticle was used as the carrier for the delivery of antroquinonol from solid-state-cultured *A. cinnamomea* extract (AC). The rats were fed with a high-fat diet and intraperitoneally injected with streptozotocin to induce diabetes. The rats were daily oral gavage by water [Diabetes (DM) and Control groups], three different doses of chitosan-silicate nanoparticle of antroquinonol from solid-state-cultured *A*. *cinnamomea* (nano-SAC, NAC): (DM+NAC1x, 4 mg/kg of body weight; DM+NAC2x, 8 mg/kg; and DM+NAC5x, 20 mg/kg), solid-state-cultured AC (DM+AC5x, 20 mg/kg), or metformin (DM+Met, 200 mg/kg) for 7 weeks.

**Results:**

The nano-SAC size was 37.68±5.91 nm, the zeta potential was 4.13±0.49 mV, encapsulation efficiency was 79.29±0.77%, and loading capacity was 32.45±0.02%. The nano-SAC can improve diabetes-induced reproductive dysfunction by regulating glucose, insulin, and oxidative enzyme and by increasing the level of testosterone, follicle-stimulating hormone, luteinizing hormone, and sperm count as well as sperm mobility. In testicular histopathology, the seminiferous tubules of *A. cinnamomea*-supplemented diabetic rats showed similar morphology with the control group.

**Conclusion:**

The nanoparticle of antroquinonol from *Antrodia cinnamomea* can be used as an effective strategy to improve diabetes-induced testicular dysfunction.

## Introduction

Metabolic syndrome is a group of health problems for a combination of dysglycemia, raised blood pressure, elevated triglyceride levels, low high-density lipoprotein cholesterol levels, and abdominal obesity. It is associated with a substantially increased risk of cardiovascular disease.^1^ Diabetes is a chronic disease that occurs either the pancreas does not produce enough insulin or the body cannot effectively use the insulin (insulin resistance, IR) and resulting high blood sugar (hyperglycemia).^2^,^3^ Oxidative stress has a positively correlation with IR and contributes to diabetes. More than 90% of diabetic patients are suffering from type-2 diabetes mellitus. Besides β-cell failure, the major pathophysiological event contributing to the development of type-2 diabetes mellitus is the resistance of target tissues to insulin.^4^ Additionally, previous study also reported that hyperglycemia increases the proinflammatory level in diabetic patient.^5^ Organs damaged, including testis, pancreas, and brain has been reported a positive association with a diabetic condition and showed adverse effects on the reproductive function.^6^,^7^ Diabetic condition also reported low essential reproductive hormones, such as luteinizing hormone, follicle stimulating hormone, and testosterone.^8^,^9^ Several oral agents have been used for diabetic treatment, such as thiazolidinedione, alpha-glucosidase inhibitors, dipeptidyl peptidase-4 inhibitors, sulfonylureas, and meglitinides.^10^,^11^ However, some anti-diabetic agents reported that it increased cardiovascular and gastrointestinal diseases prevalence.^12^,^13^ Therefore, the investigation of an alternative anti-diabetic agents with less adverse effects is a major topic for future research for example using natural products or though functional foods.

*Antrodia cinnamomea* (also known as *A. camphorata*) is a well-known mushroom that has been used as herbal medicine and known as “ruby in mushroom” for centuries in Taiwan. This mushroom has been used in the treatment of various diseases, such as diarrhea, abdominal pain, hypertension, anticancer, and antitumor activities as well as immunosuppressive effect.^14^–^18^ Due to the growth rate of natural *A. cinnamomea* in the wild is very slow, difficult to cultivate, and expensive to obtain fruiting bodies; cultivation techniques have been developed, including solid-state fermentation for mycelia of *A. cinnamomea*.^19^–^21^ *A. cinnamomea* contains large amounts of natural ingredients, such as triterpenoids, benzenoids, lignans, benzoquinone derivatives, succinic and maleic derivatives, and miscellaneous compounds.^22^ A previous study reported that triterpenoid compound from petri dish-cultured *Antrodia cinnamomea* ameliorates male reproductive function in rat model.^23^ A previous study reported that besides the triterpenoids and polysaccharides, antroquinonol also present in solid-state fermentation of mycelium of *A. cinnamomea*.^24^ It also can be extracted by ethanolic extraction from mycelium of *A. cinnamomea*.^25^ Antroquinonol is an ubiquinone derivates from miscellaneous compounds.^26^ Several studies reported that solid-state fermented mycelium of *A. cinnamomea* possessed anticancer activity and immunosuppressive effects.^18^,^26^,^27^ It also attenuated the progression of nephritis in systemic lupus mice model and modulated atherosclerosis development.^28^,^29^ Additionally, a synthetic antroquinonol improved insulin resistance by triggering adenosine monophosphate-activated protein kinase and also inhibiting dipeptidyl peptidase IV activities.^30^ However, besides its potential effects, antroquinonol (a kind of ubiquinones) is lipid-soluble and has been reported that its poor aqueous solubility and relatively low bioavailability through oral delivery system.^31^,^32^ Therefore, various mechanisms have been formulated to enhance ubiquinones utilization include nanoparticle form.^31^,^33^

Nanotechnology has been used to enhance the bioavailability of poorly soluble material. Whereas, silica and chitosan have been reported by various previous studies relate their ability to enhance the bioavailability of some low soluble materials, such as triterpenoids and curcumin.^23^,^34^ Therefore, we hypothesized that the nanoparticle form of antroquinonol enhances its utilization. As such, this study aimed to characterize the chitosan-silicate nanoparticle of antroquinonol-rich extract from solid-state-cultured *A. cinnamomea* (nano-SAC) and to investigate its ameliorative effects on male reproduction function in high-fat diet and streptozotocin-induced type-2 diabetic rat model.

## Materials and Methods

### Materials

Solid-state fermented *Antrodia cinnamomea* mycelium ethanol extract (SAC) was provided by Taipei Lantyng Biotechnology Co., Ltd. (Taipei, Taiwan). This ethanolic extract composed of antroquinonol (3.604 mg/mL), 4,7-dimethoxy-5-methyl-1,3-benzodioxole (0.063 mg/mL), and β-glucan (10.97%, w/w). The 1,1,3,3-tetramethoxypropane (Malonaldehyde, MDA), dimethyl sulfoxide (DMSO), heparin, 1, 1-diphenyl-2-picrylhydrazyl (DPPH), pancreatin, pepsin, peroxidase, streptozotocin (STZ), and trolox were purchased from Sigma-Aldrich (St. Louis, Missouri, USA). Chitosan powder was purchased from Lytone Enterprise (Taipei, Taiwan). Roswell Park Memorial Institute (RPMI) medium was purchased from Gibco (Carlsbad, California, USA). The aspartate transaminase (AST), alanine transaminase (ALT), creatinine, blood urea nitrogen (BUN), superoxide dismutase (SOD), and glutathione peroxidase (GPx) kits were purchased from Randox Laboratories Ltd. (Ardmore, Colorado, UK). The blood glucose and insulin kits were purchased from Mercodia (Uppsala, Sweden). The follicle stimulating hormone (FSH), luteinizing hormone (LH), and testosterone ELISA kits were purchased from Abcam (New Haven, Connecticut, USA).

### Preparation of *Antrodia cinnamomea* Encapsulated Silica–Chitosan Nanoparticles (Nano-SAC)

Nano-SAC were prepared by using sodium silicate and chitosan solution.^35^ Sodium silicate was dissolved in 30 mL buffer (0.05 M sodium acetate) to prepare 0.55% (w/w) solution (pH 6.0). Immediately after the dissolution of silicate on a magnetic stirrer, 6 mL of *A. cinnamomea* was dissolved in ethanol (0.1%, w/w) and 3 mL chitosan was dissolved in acetic acid (0.55%; w/w) solution (pH 5.6). The solution containing chitosan, silicate, and *A. cinnamomea* were mixed completely. The suspensions were centrifuged at 12,000 *×*g (High-speed centrifuge, Hettich CR-12, Hamburg, Germany) for 30 min. The supernatant was collected to new collection tube and then dried by using a freeze dryer (Freeze drying system FD4.5, Kingmech, Taipei, Taiwan).

### Nano-SAC Characterizations

For morphology and shape analysis, nanoparticle suspension was dropped on an aluminum foil slide, dried at oven (37°C) and observed under a scanning electron microscope (SEM) (Hitachi S-4800, Tokyo, Japan) at an acceleration voltage of 10 kV. Prior to imaging, samples were sputtered. Coated with a thin layer of gold using a Hitachi sputter coater (Model-E1010 Ion sputter) under vacuum.^35^ The zeta potential of nanoparticles were measured by using Zetasizer Nano ZS (Malvern Instrument, Worcester-shire, UK).^36^

### Encapsulation Efficiency and Loading Capacity Analysis

The nano-SAC was dissolved in methanol for measuring *A. cinnamomea* encapsulation efficiency (EE) and loading capacity (LC) and filtered by a using 0.22 µm microporous membrane. The amount of *A. cinnamomea* in the solution was determined by high-performance liquid chromatography (HPLC, JASCO, Eaton, Maryland, USA). HPLC detection was performed using a C18 column (100 Å, 5 µm, 4.6mm × 250 mm, Waters, Massachusetts, USA). The mobile phase, consisting of methanol and deionized distilled water (60:40) (v/v), was maintained at a flow rate of 1.0 mL/min. The ultraviolet detector wavelength was 254 nm, and the injection volume was 20 µL.^36^,^37^ The EE (%) and LC (%) were calculated according to the formula:

$${\rm{EE}}\left(\rm \% \right){\rm{ = }}{\mkern 1mu} {{\matrix{ \matrix{
{\rm{Total}}\,{\rm{amount}}\,{\rm{of}} \hfill \cr \,{\rm{A}}.{\rm{cinnamomea}} \hfill \cr} & {\rm{ - }} & \matrix{
{\rm{free \ A}}.{\rm{cinnamomea}} \hfill \cr {\rm{in}}\,{\rm{the}}\,{\rm{supernatant}} \hfill \cr} \cr } } \over \matrix{ {\rm{Total}}\,{\rm{amount}}\,{\rm{of}}\, \hfill \cr {\rm{A}}.{\rm{cinnamomea}} \hfill \cr} }$$

$${\rm{LC}}\left(\rm \% \right){\rm{ = }}{{\matrix{ \matrix{ {\rm{Total}}\,{\rm{amount}}\,{\rm{of}}\, \hfill \cr {\rm{A}}.{\rm{cinnamomea}} \hfill \cr} & {\rm{ - }} &\matrix{ {\rm{free}}\,{\rm{A}}.{\rm{cinnamomea}}\, \hfill \cr {\rm{in}}\,{\rm{the}}\,{\rm{supernatant}} \hfill \cr} \cr } } \over \matrix{ {\rm{Wieght}}\,{\rm{of}}\,{\rm{the}}\, \hfill \cr {\rm{nanoparticles}} \hfill \cr} }{\rm{x100}}\rm \% $$

### Fourier Transform-Infrared (FT-IR) Spectroscopy Analysis

The Fourier-transform infrared (FTIR, Bruker-Tensor II, Massachusetts, USA) experiments were conducted using by potassium bromide (KBr) disc method. Each spectrum was analyzed in the range of resolution from 400 to 4000 cm^−1^ and a total of 16 scans were performed.^36^

### Animal Experiment

Forty two of 5-week-old male Sprague–Dawley (SD) rats were purchased from the BioLASCO Taiwan Co., Ltd. (Yilan, Taiwan). Each rat was housed individually in disinfectant stainless-steel cages under controlled temperature (23±2ºC) and humidity (40–60%) with 12 h light/12 h dark cycle. The food and water were provided *ad libitum*. The rats were domesticated for a week and given standard chow-fed diet (Laboratory Rodent Diet 5001). All procedures followed the standard of Institutional Animal Care and Use Committee (IACUC Approval No. 107001) of the National Taiwan Ocean University, Taiwan.

**Figure 1:**
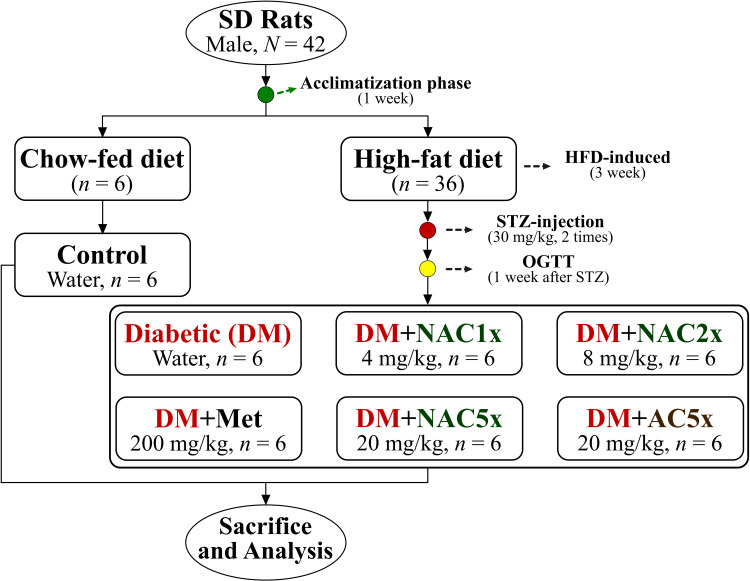
The flowchart of nano-SAC treatment in high-fat diet and streptozotocin-induced diabetic rats.

Briefly, after acclimatization phase the rats were divided into 2 main groups, they are normal control group (*n* = 6) and diabetic group (n = 36). The normal control (Control) group was still fed with standard chow-fed diet (CFD), whereas diabetic group was fed with a high-fat diet (HFD, 40% calories made by adding lard). After being fed with the HFD for 3 weeks, the diabetic group rats were intraperitoneal injection with streptozotocin (STZ, 35 mg/kg; two times each interval of 1 week) to induce diabetic model. A previous study reported that fed with an HFD and a low-dose of STZ injection were followed to induce type-2 diabetic in the rat model.^38^ After that, the diabetic rats were divided into 6 groups (n = 6), they are diabetic control rat was daily oral gavage with distilled water (DM); diabetic rat with metformin (DM+Met, 200 mg/kg) was taken as the positive control; diabetic rats were administrated by oral gavage of nano-SAC (4 mg/kg, DM+NAC1x; 8 mg/kg, DM+NAC2x; and DM+NAC5x, 20 mg/kg) according to Sudirman et al^23^ and diabetic rat was administrated *A. cinnamomea* alone (DM+AC5x, 20 mg/kg) as shown in Figure 1. The rats were sacrificed after treatment for 7 weeks. The rats have fasted prior to sacrifice for 12 h. The whole blood and organs were collected for future analysis.

### Blood Sample Collection

The whole blood samples were obtained by using heparinized-syringe at the cardiac puncture were centrifuged at 1750 *×*g for 15 min at 4°C. The supernatant (plasma) was collected and directly used or kept at −80°C for future analysis.^39^

### Oxidative Stress Analysis

The superoxide dismutase (SOD) and glutathione peroxidase (GPx) enzymatic antioxidants were analyzed by using commercial kits. The lipid peroxidation in terms of malonaldehyde (MDA) formation in plasma was measured according to the previous method.^40^ Briefly, plasma was mixed with the reactive solution (15% [w/v] trichloroacetic acid and 0.375% [w/v] thiobarbituric acid in 0.25 n-HCl). The mixture was added to 300 µL n-butanol and centrifuged at 1500 *×*g for 10 min. The supernatant absorbance was immediately measured at the 532 nm by using a spectrophotometer.

### Sperm Count and Motility Analysis

Sperm preparation and analysis was performed as described by the previous method.^41^ Briefly, the epididymides of rat were dissected, removed and the caudal epididymis was minced in 5 mL of pre-warmed RPMI medium to allow spermatozoa to leave the epididymal tubules and incubated for 30 min at 37°C. The percentage of motile spermatozoa and abnormality were recorded under a light microscope and counted by using a hemocytometer.

### Testis Homogenized Preparation and Histopathological Analysis

From each rat, one testis was stored at −80°C for future analysis and the other one was soaked in 4% formaldehyde solution. The supernatant of homogenized testis was prepared by following previous study.^42^ Briefly, the samples were washed for three times in phosphate buffer saline (PBS, pH 7.4) to remove host material and stored at −20°C. After thawing, the samples were homogenized in homogenizing buffer (2 mL, PBS) by using a glass homogenizer (Hitachi SCR 20BA, Tokyo, Japan). Then the suspension was centrifuged (10,000 *×*g for 30 min at 4°C) and the supernatant was stored at −20°C for future analysis. And the other one for histopathological analysis. Briefly, five-micrometer-thick paraffin sections of testis were cut and send to Rapid Science Co., Ltd., for hematoxylin and eosin (H&E) staining.

### Statistical Analysis

Data were expressed as mean ± standard deviation (SD). Statistical analysis by Duncan’s multiple range tests (*P*<0.05) were conducted by a using Statistical Package for the Social Sciences (SPSS v22.0, IBM Corporation, Armonk, NY, USA) software to analyze the experimental data.

## Results and Discussion

### Characteristics of Nano-SAC

**Figure 2:**
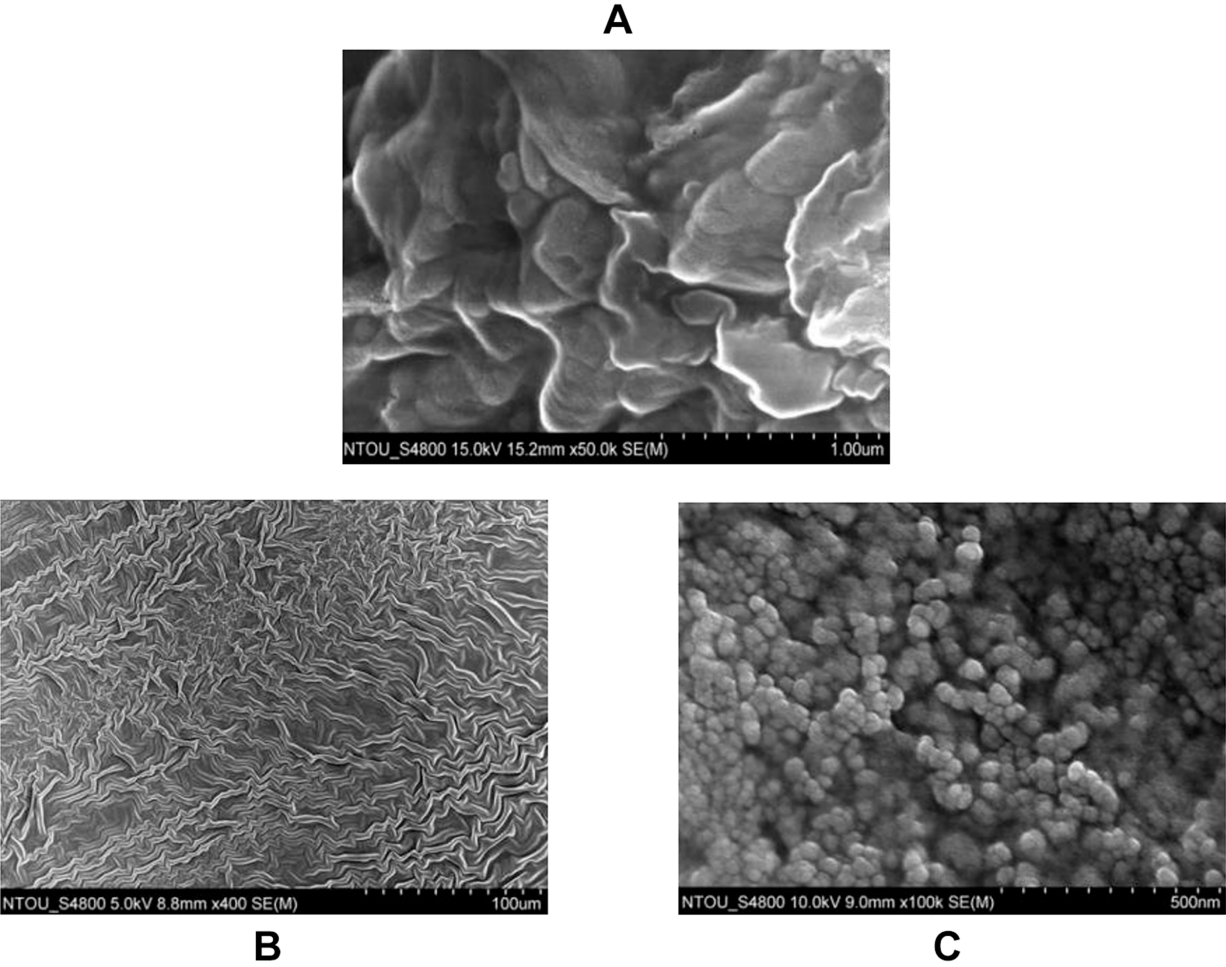
The morphology of (**A**) Solid-state-cultured *A. cinnamomea* (SAC) extract, (**B**) chitosan with silicate, and (**C**) nano-SAC as showed by scanning electron microscopy (SEM). **Abbreviations:** AC, *A. cinnamomea*; DM, diabetes group; Met, metformin; NAC, nano-SAC; n, number of samples each group; SAC, solid-state-cultured *A. cinnamomea*.

The morphology of nano-SAC was investigated by scanning electron microscope (SEM) without further purification process. The nano-SAC showed a spherical in shapes and tendency to form a “grape-like morphology,” as shown in Figure 2. Whereas, the particle size was 37.68±5.91 nm by SEM observation (Table 2). A previous study also reported similar shape and form in the nanoparticle of triterpenoids from Petri dish-cultured *Antrodia cinnamomea* (nano-PAC). The result showed smaller particle size when compared nano-PAC and a high percentage of encapsulation efficiency (EE).^23^ This study also showed smaller particle size and high in EE percentage when compared to silica-chitosan and *A. camphorata* extract (ACE) polysaccharides encapsulation.^43^ The environmental condition and synthesis process were effected to the nanoparticle shape and size, such as polymer concentration and molecular weight, surface tension, acid condition, and conductivity.^44^ Therefore, we hypothesized that electrostatic interaction between silica, chitosan, and SAC, as well as the fabrication process involved in the size and form of nano-SAC.

**Table 1:** The Characteristics of Nano-SAC

Parameter	Size (nm)	Zeta Potential (mV)	EE (%)	LC (%)
Nano-SAC	37.68±5.91	4.13±0.49	79.29±0.77%	32.45±0.02

**Notes:** Data are shown as mean ± SD (n = 3). The nanoparticle size was measured by SEM. **Abbreviations:** EE, encapsulation efficiency; LC, loading capacity; SEM, scanning electron microscope.

**Table 2:** The Fasting Plasma Glucose, Insulin, and HOMA-IR Levels in Diabetic Rats After 7 Weeks

Parameter	Control	Diabetes	DM+Met	DM+NAC1x	DM+NAC2x	DM+NAC5x	DM+AC5x
FPG (mg/dL)	102.58±8.11^c^	264.44±5.85^a^	118.41±4.58^bc^	155.67±6.99^b^	137.76±8.00^b^	108.61±6.18^c^	176.79±8.11^a^
Insulin (ng/L)	0.12±0.01^b^	0.28±0.11^a^	0.14±0.04^b^	0.19±0.05^ab^	0.15±0.04^b^	0.14±0.12^ab^	0.20±0.12^ab^
HOMA-IR	1.09±0.27^c^	4.38±1.97^a^	1.23±0.32^c^	2.08±0.55^bc^	1.52±0.41^c^	1.08±0.61^c^	2.02±1.35^bc^

**Notes:** Data are shown as the mean ± SD (n = 6). The values with different letters (a–c) represent significantly (*P*<0.05) different as analyzed by Duncan’s multiple range test. The homeostasis model assessment−estimated insulin resistance (HOMA-IR) = fasting plasma glucose (mmol/L) × fasting plasma insulin (mU/L)/22.5. **Abbreviations:** DM, diabetes group; DM+Met, diabetes + 200 mg/kg of metformin; DM+NAC1x, diabetes + 4 mg/kg of nano-SAC; DM+NAC2x, diabetes + 8 mg/kg of nano-SAC; DM+NAC5x, diabetes + 20 mg/kg of nano-SAC; DM+AC5x, diabetes + 20 mg/kg of SAC.

The nanoparticle (NP) size, shape, surface charge, and surface coating affected cellular uptake of the nanoparticle.^45^,^46^ A high ratio of surface area-to-volume with a smaller size make nanoparticle easier to release the encapsulated drug from the nanoparticle via diffusion.^47^ Additionally, with the smaller size, nanoparticles also will easy to enter the cell.^48^ Nanoparticle with positive surface charge also shows greatest cellular update when compared to other charges.^49^ As shown in Table 1, the zeta potential of the nano-SAC was about 4.13±0.49 mV. Additionally, a previous study also showed that nanoparticle with positive charge improves the drug delivery efficiency.^50^ Also, gold nanoparticles with positive charge showed more stability and interacted strongly with the capillary wall than negative charged NP.^51^ Along with surface changed (zeta potential) values of NP, other factors, such as solution chemistry, presence of surfactants, and also material properties, affect the NP’s physical stability.^52^

The nano-SAC also presented high encapsulation efficiency (EE, 79.29±0.77%) when compared to previous studies. For example, silica-chitosan nanoparticle form with *A. cinnamomea* triterpenoids and *A. cinnamomea* extract (ACE) polysaccharides possess a percentage of EE were 73.35% and 63.5%, respectively.^23^,^35^ Whereas, EE is the percentage of bioactive compound (antroquinonol) which successfully entrapped by the nanoparticle.^53^ As such, EE is one of the important parameter for drug delivery system or active ingredient, especially for expensive drugs. Whereas, nanoparticles have been reported to their potential for drug delivery system enhancement.^47^,^54^

This present study successfully entrapped antroquinonol-rich extract from *A. cinnamomea* in silica-chitosan nanoparticles. The silica-chitosan nanoparticles also successfully deliver triterpenoid and polysaccharide extracts from *A. cinnamomea*.^23^,^35^ A previous study reported that silica nanoparticles as drug delivery show various advantages, such as easy modification of the surface and large surface area. Whereas, chitosan also has been reported as drug carrier agent due to some benefits, such as biodegradable, pH-responsive, high loading capacity, and biocompatibility.^55^,^56^

### Functional Groups of Nano-SAC

**Figure 3:**
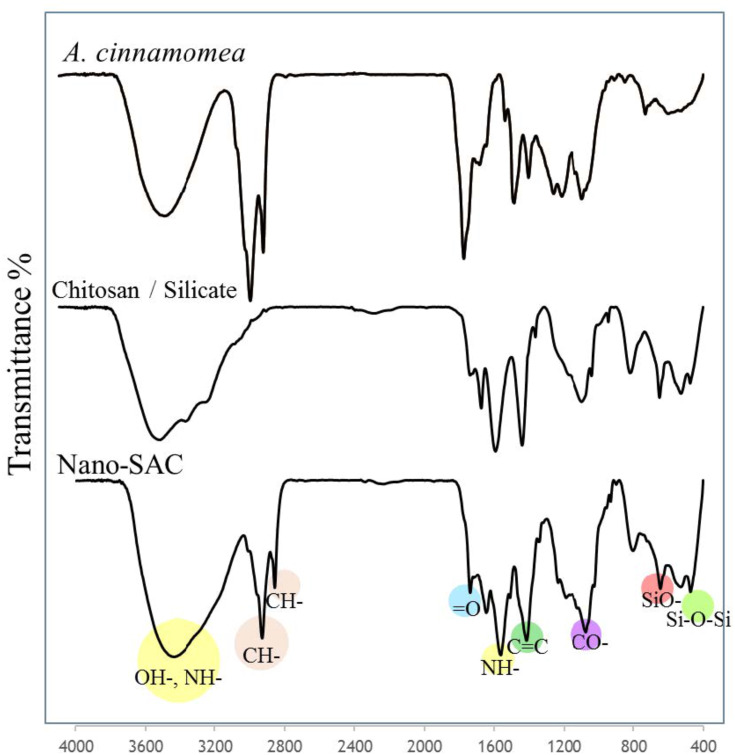
The spectrum of *Antrodia cinnamomea* extract, chitosan with silicate, and nano-SAC as measured by Fourier transform-infrared spectroscopy. **Abbreviation:** nano-SAC, nanoparticle form of silica-chitosan and solid-state-cultured *A. cinnamomea*.

Fourier-transform infrared (FT-IR) spectroscopy was used to examine the presence of chitosan, silicate, and *A. cinnamomea*. Chitosan with silicate exhibited the basic characteristic peaks at 3440 cm^−1^ (OH- stretch and NH- stretch overlapping), 2926 cm^−1^ and 2863 cm^−1^ (asymmetric and symmetric stretching of CH-, respectively), 1600–1700 cm^−1^ (=O stretch for amide), 1550 cm^−1^ (NH- bend deformation), 1414 cm^−1^ (C=C stretch), 1070 cm^−1^ (CO- stretch), 660 cm^−1^ (SiO- stretch), 500–450 cm^−1^ (Si–O–Si stretch).^36^,^57^,^58^ The absorption peaks characteristic of both silica and chitosan appeared in the silica-chitosan nanoparticle spectrum as shown in Figure 3.

### Nano-SAC Improves Glucose, Insulin, and HOMA-IR Levels in vivo Model

The effect of the nano-SAC (NAC) on fasting plasma glucose (FPG), insulin and homeostasis model assessment-estimated insulin resistance (HOMA-IR) levels in normal and diabetic rats were shown in Table 2. The FPG and insulin level in untreated diabetic (DM) rats were significantly elevated 2.5-fold and 2-fold, respectively as compared with those of control rats after treatment for 7 weeks. Metformin and nano-SAC significantly altered the changes of FPG and insulin levels, the DM+NAC5x was even found to be more effective than metformin. The DM rats showed a significant elevation of HOMA-IR that was decreased significantly upon administration of metformin, nano-SAC, and SAC alone. Whereas, high-dose of nano-SAC (NAC5x) more effective to ameliorate the HOMA-IR level. The insulin resistance is defined by HOMA-IR.^59^,^60^

### Nano-SAC Ameliorates Oxidative Stress in Diabetic Rats

Superoxide dismutase (SOD) and glutathione peroxidase (GPx) were major enzymatic antioxidants in the human body. In Table 3, the untreated diabetic (DM) rats showed less plasma SOD and GPx activities when compared to the control group. However, after treated with nano-SAC, metformin, and SAC alone resulted in a significant recovery in the SOD activity, and there is no significant effect on GPx activity when compared to untreated diabetic rats. SOD and GPx were known as the primary antioxidants responsible for maintaining the optimum reactive oxygen species (ROS) level.^61^–^63^ Diabetes is a degenerative disease that has deleterious effects on male reproductive function, possibly through an increase in oxidative stress. *Antrodia cinnamomea* is known to have anti-aging, anti-cancer, anti-diabetic, anti-inflammatory, and antioxidant.^23^,^64^ A previous study reported that antroquinonol reduced oxidative stress by enhancing the nuclear factor E2-related factor 2 (Nrf2) signaling.^65^ *Antrodia cinnamomea* extract also exhibited antioxidant capacity by 1,1-diphenyl-2-picrylhydrazyl (DPPH) free radical scavenging and superoxide radical scavenging (SOD) assay.^66^ Additionally, ethanolic extract of *A. cinnamomea* nanoparticles reduced levels of nitric oxide (NO) and superoxide anion (O_2_^−^) in Leydig (LC-540) cells.^23^ According to these conditions, antroquinonol could improve reproductive function by improving oxidative stress. A previous study reported that oxidative stress has been recognized as one of the risk factors of male reproductive dysfunction.^67^

**Table 3:** Activities of SOD, GPx, and MDA Level of Plasma in STZ-Induced Diabetic Rat After Treatment 7 Weeks

Parameter	Control	Diabetes	DM+Met	DM+NAC1x	DM+NAC2x	DM+NAC5x	DM+AC5x
SOD (U/mL)	51.95±1.06^a^	32.45±2.19^d^	50.21±1.98^a^	43.03±2.26^c^	45.98±3.00^bc^	47.27±2.63^b^	47.32±1.78^b^
GPx (U/mL)	37.34±9.62^a^	36.87±4.55^b^	21.12±5.45^a^	25.67±3.61^ab^	30.58±6.10^ab^	28.09±3.69^ab^	29.33±6.54^ab^
MDA (nmol/mL)	1.78±0.23^d^	3.30±0.06^a^	2.11±0.28^c^	2.73±0.15^b^	2.49±0.18^bc^	2.09±0.14^c^	2.27±0.28^bc^

**Note:** Data are shown as the mean ± SD (n = 6). The values with different letters (a–d) represent significantly different (*P*<0.05) as analyzed by Duncan’s multiple range test. **Abbreviations:** DM, diabetes group; DM+Met, diabetes + 200 mg/kg of metformin; DM+NAC1x, diabetes + 4 mg/kg of nano-SAC; DM+NAC2x, diabetes + 8 mg/kg of nano-SAC; DM+NAC5x, diabetes + 20 mg/kg of nano-SAC; DM+AC5x, diabetes + 20 mg/kg of SAC.

The triterpenoids of *A. cinnamomea* nanoparticles showed a high of LC-540 cell viability (≥ 90%).^23^ A previous study also reported that *A. cinnamomea* extracts polysaccharides nanoparticles showed non-toxic to normal cell lines.^35^ According to these studies, we hypothesis that the antroquinonol of *A. cinnamomea* nanoparticle also possessed similar effects, especially the case of its toxicity in the cell-based study. Additionally, the antroquinonol of *Antrodia camphorata* extract had no toxicity effects both in vitro and in vivo assay.^68^

Malondialdehyde (MDA) was very useful for evaluating lipid peroxidation.^69^ The cytosolic fractions of the untreated diabetic (DM) rat plasma also had significantly more MDA level than the normal as shown in Table 3. The DM+Met and DM+NAC5x led to a recovery in plasma MDA levels close to control levels. Nano-SAC inhibited the diabetes-induced oxidative stress by increasing the activities of SOD and glutathione peroxidase as well as reducing MDA levels. Lipid peroxidation is the process of oxidation in lipids and finally results in cell damage. MDA is produced as the result of lipid peroxidation of polyunsaturated fatty acids.^70^

### Effects of Nano-SAC on the Reproductive Hormones

**Figure 4:**
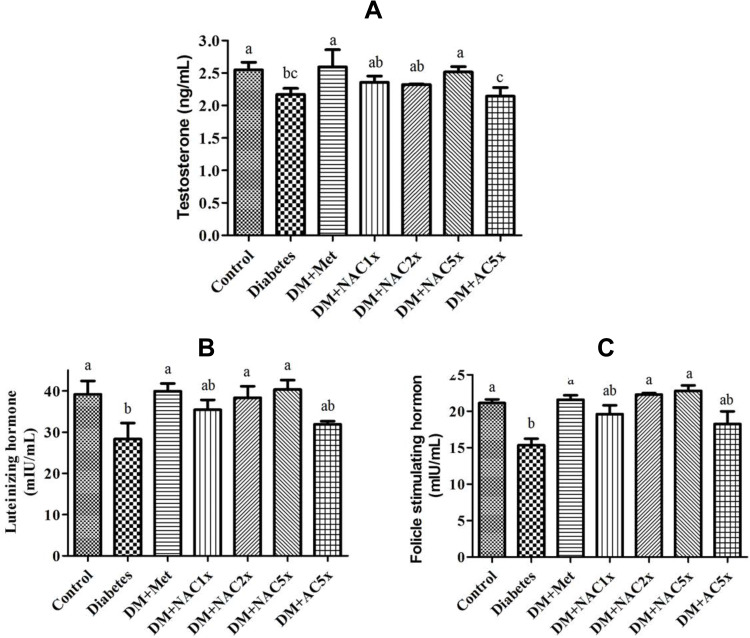
Expression of (**A**) testosterone hormone, (**B**) luteinizing hormone, and (**C**) follicle-stimulating hormone in plasma of Streptozotocin-induced diabetic rat after treatment 7 weeks. **Notes:** Data are shown as the mean ± SD (n = 6). The values with different letters (a–c) represent significantly different (*P*<0.05) as analyzed by Duncan’s multiple range test. **Abbreviations:** DM, diabetes group; DM+Met, diabetes + 200 mg/kg of metformin; DM+NAC1x, diabetes + 4 mg/kg of nano-SAC; DM+NAC2x, diabetes + 8 mg/kg of nano-SAC; DM+NAC5x, diabetes + 20 mg/kg of nano-SAC; DM+AC5x, diabetes + 20 mg/kg of SAC.

The hormonal study revealed that the plasma luteinizing hormone (LH), follicle-stimulating hormone (FSH), and testosterone levels decreased in the untreated diabetic (DM) group compared with the control group (Figure 4). After given with metformin and nano-SAC showed that plasma LH, FSH, and testosterone levels were considerably increased and closed to the control group. However, SAC does not shown significant differences with the DM group. According to this condition, nanoparticle form of SAC (nano-SAC) increased the bioavailability of SAC. According to these data, we hypothesized that antroquinonol nanoparticles were also upregulating the upstream regulator of gonadal hormones (such as kisspeptin protein in hypothalamus) and resulting in improved male reproductive dysfunction. The hypothalamus-pituitary-gonadal (HPG) axis regulates the different stages of reproductive activities, such as sexual behavior, spermatogenesis, and fertility.^71^ The FSH secreted by the Sertoli cells plays a vital role in testicular development. The LH released from the pituitary gland subsequently binds to the testicular Leydig cells through LH and then activates the biosynthesis of testosterone.^72^ The present data (Table 3) also showed that high-dose of antroquinonol nanoparticles reduce the plasma glucose with the value similar to normal conditions. The adverse effects of diabetes on male reproductive functions might be mediated through hormonal alterations in the HPG axis on testicular and sperm cells.^73^

### Effect of Nano-SAC on the Sperm Count and Motility

**Figure 5:**
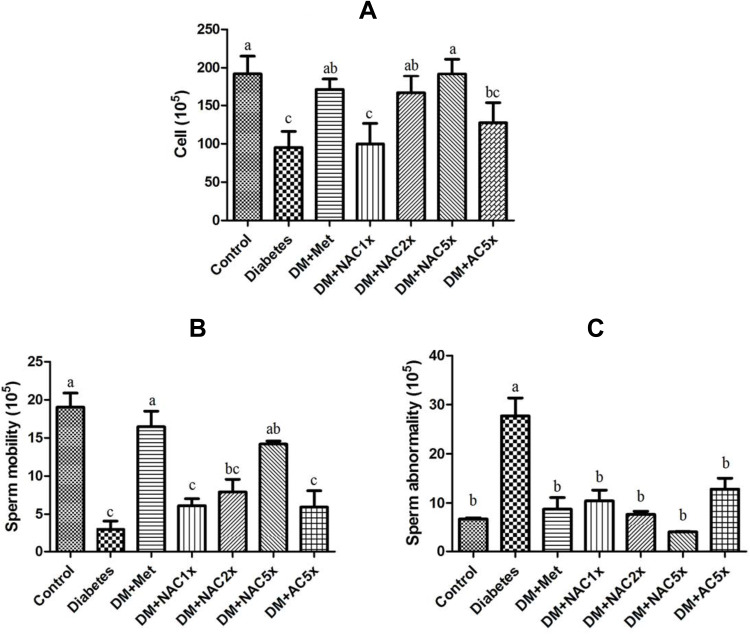
(**A**) Sperm count, (**B**) sperm mobility, and (**C**) sperm abnormality in STZ-induced diabetic rats after treatment 7 weeks. **Notes:** Data are shown as the mean ± SD (n = 6). The values with different letters (a–c) represent significantly different (*p*<0.05) as analyzed by Duncan’s multiple range test. **Abbreviations:** DM, diabetes group; DM+Met, diabetes + 200 mg/kg of metformin; DM+NAC1x, diabetes + 4 mg/kg of nano-SAC; DM+NAC2x, diabetes + 8 mg/kg of nano-SAC; DM+NAC5x, diabetes + 20 mg/kg of nano-SAC; DM+AC5x, diabetes + 20 mg/kg of SAC.

In Figure 5A and B, the diabetic rats showed significantly less sperm count and sperm motility than the control group. Treatment with metformin and nano-SAC5x recovered motility and sperm count similar to control group, nano-SAC had even dose-dependent changed. In Figure 5C, DM has high abnormality than the control group. Even though in metformin and nano-SAC had no significant differences in these group, nano-SAC still had dose-dependent changed. The recovery of sperm parameters of nano-SAC and SAC rats can be attributed to antioxidant properties of *A. cinnamomea*. Whereas, nano-SAC has shown protective effects more effectively against varieties of chemicals-induced decreases in sperm parameters. A previous study reported that *A. cinnamomea* extract and metabolite showed scavenging effects against free radicals.^66^,^74^ On the other hands, DM+Nano (chitosan with silicate) does not shown significant differences with the diabetes group.^23^

### Liver and Kidney Functions

Enzyme activities of alanine transferase (ALT), aspartate transferase (AST), creatinine, and blood urea nitrogen (BUN) levels of type-2 diabetes rats showed a general increase as compared to their control groups. The liver is the central metabolic organ in the body which is responsible for glucose and lipid homeostasis.^39^ In the present study, the rise in the activity of ALT was thought because of the hepatocellular damage accompanied by a rise in AST. An increase in the activities of AST and ALT in the liver of diabetic animals has been frequently reported.^75^ In Table 4, the activity of ALT significantly increased in diabetes group compared to the control group, and after gave nano-SAC for 7 weeks, it was reduced significantly.

**Table 4:** Plasma ALT, AST, Creatinine, and BUN in Diabetic Rats After Treatment 7 Weeks

Parameter	Control	Diabetes	DM+Met	DM+NAC1x	DM+NAC2x	DM+NAC5x	DM+AC5x
AST (U/L)	9.85±1.55^a^	13.36±1.67^b^	11.52±0.93^ab^	14.14±1.85^b^	12.83±1.23^ab^	10.83±1.26^ab^	14.28±1.15^b^
ALT (U/L)	8.16±1.29^a^	15.35±1.90^c^	9.25±1.59^a^	14.96±1.66^c^	10.91±1.32^ab^	9.88±2.05^a^	13.24±1.47^bc^
Creatinine (mg/dL)	0.53±0.12^a^	0.85±0.10^c^	0.61±0.07^ab^	0.72±0.11^bc^	0.62±0.16^ab^	0.56±0.11^ab^	0.80±0.09^c^
BUN (mg/dL)	20.90±2.29^a^	42.97±3.03^c^	27.02±3.80^b^	39.80±2.95^bc^	33.89±3.61^b^	27.54±2.76^b^	26.73±4.26^b^

**Note:** Data are shown as the mean ± SD (n = 6). The values with different letters (a–c) in same rows represent significantly different (*P*<0.05) as analyzed by Duncan’s multiple range test. **Abbreviations:** ALT, alanine transferase; AST, aspartate transferase; BUN, blood urea nitrogen; DM, diabetes group; DM+Met, diabetes + 200 mg/kg of metformin; DM+NAC1x, diabetes + 4 mg/kg of nano-SAC; DM+NAC2x, diabetes + 8 mg/kg of nano-SAC; DM+NAC5x, diabetes + 20 mg/kg of nano-SAC; DM+AC5x, diabetes + 20 mg/kg of SAC.

In Table 4, the creatine and BUN levels in the diabetes group was significantly higher than the control group, and after gave nano-SAC for 7 weeks, it had significant reduced compared to diabetes. Creatinine is a chemical waste product produced from creatinine during muscle metabolism. The kidneys filter out most of the creatinine before disposing of through urine. As a consequence of the way in which creatinine is excreted by the kidney, creatinine measurement is used almost exclusively in the assessment of kidney function. Creatinine is regarded as the most useful endogenous marker in the diagnosis and treatment of kidney disease.^76^,^77^ BUN is synthesized in the liver from ammonia, as a result of deamination of amino acids. This biosynthetic pathway is the chief means of the excretion of surplus nitrogen by the body. Measurements obtained by this test are used in the diagnosis of renal and metabolic disorders.^77^,^78^

### Effect of Nano-SAC on the Testis Histopathological Structure

**Figure 6:**
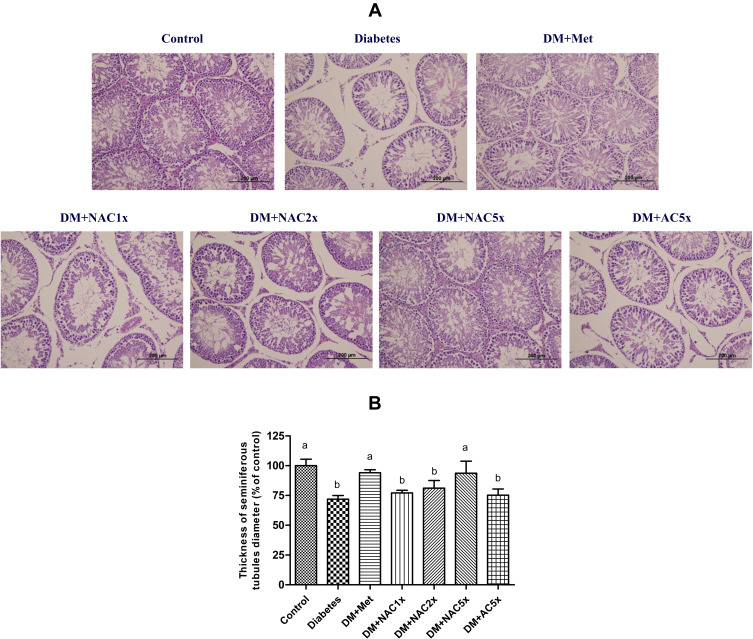
The seminiferous tubules (**A**) morphology and (**B**) thickness of diameter with hematoxylin and eosin staining and of STZ-induced diabetic rats after treatment for 7 weeks. **Notes:** Data are shown as the mean ± SD (n = 6). The values with different letters (a–b) represent significantly different (*p*<0.05) as analyzed by Duncan’s multiple range test. **Abbreviations:** DM, diabetes group; DM+Met, diabetes + 200 mg/kg of metformin; DM+NAC1x, diabetes + 4 mg/kg of nano-SAC; DM+NAC2x, diabetes + 8 mg/kg of nano-SAC; DM+NAC5x, diabetes + 20 mg/kg of nano-SAC; DM+AC5x, diabetes + 20 mg/kg of SAC.

The testes showed normal histological structure of the seminiferous tubules and normal spermatogenesis in the control group (Figure 6). Whereas, the untreated diabetic (DM) group displayed morphological alterations with diminished seminiferous tubule sizes and diameters, degenerated spermatogonia, and disappeared spermatids in the seminiferous tubular lumen, which were similar to consistent with literature reports.^79^,^80^ The administration with metformin and high-dose of nano-SAC significantly inhibited the above said phenomenon induced by diabetes and thereby prevented the reduction in size, diameter, and number of seminiferous tubules as well as number of spermatogonia, spermatocytes, and spermatids. Thus, the morphological evidence demonstrated that nano-SAC supplementation can significantly attenuate the testicular damage induced by diabetes, especially in high-dose of nano-SAC.

Overall, antroquinonol functions successfully enhanced by the nanoparticle form when compared to antroquinonol alone. Antroquinonol is characterized by low water solubility, therefore its low bioavailability, especially for the oral delivery system. Whereas, nanoparticles have been accepted to increase the oral bioavailability of the low water-soluble compounds.^81^ The nanocarriers also have been recognized in enhancing the biological and physicochemical properties of the bioactive compounds. It also more easier to take up by cells than large molecules.^82^

## Conclusions

Overall, we have successfully demonstrated that extract antroquinonol-rich extract from *Antrodia cinnamomea* with silica-chitosan in nanoparticle form (nano-SAC). The obtained nanoparticle was almost spherical and had a small size with controlled drug release ability. Oral supplementation of nano-SAC showed ameliorative effects on high-fed diet and streptozotocin-induced diabetic rats. It was observed that the nano-SAC reduced hyperglycemia and malondialdehyde level. It also improved sperm abnormality and restored sperm motility as well as sperm count. Additionally, the administration of nano-SAC improved the seminiferous tubule morphology in diabetic rats. Therefore, nano-SAC has potential as an alternative for anti-diabetic agents and developed as a functional food to enhance male reproductive function.
